# Replication in the Mononuclear Phagocyte System (MPS) as a Determinant of Hantavirus Pathogenicity

**DOI:** 10.3389/fcimb.2020.00281

**Published:** 2020-06-12

**Authors:** Martin J. Raftery, Pritesh Lalwani, Nina Lütteke, Lidija Kobak, Thomas Giese, Rainer G. Ulrich, Lukas Radosa, Detlev H. Krüger, Günther Schönrich

**Affiliations:** ^1^Institute of Virology, Charité–Universitätsmedizin Berlin, Freie Universität Berlin, Humboldt-Universität zu Berlin, Berlin Institute of Health, Berlin, Germany; ^2^Institute of Immunology, University Hospital Heidelberg, Heidelberg, Germany; ^3^Friedrich-Loeffler-Institut, Federal Research Institute for Animal Health, Institute of Novel and Emerging Infectious Diseases, Greifswald-Insel Riems, Germany

**Keywords:** hantavirus, viral pathogenesis, monocytes, dendritic cells, hantaviral entry, inflammatory DCs

## Abstract

Members of different virus families including *Hantaviridae* cause viral hemorrhagic fevers (VHFs). The decisive determinants of hantavirus-associated pathogenicity are still enigmatic. Pathogenic hantavirus species, such as Puumala virus (PUUV), Hantaan virus (HTNV), Dobrava-Belgrade virus (DOBV), and Sin Nombre virus (SNV), are associated with significant case fatality rates. In contrast, Tula virus (TULV) only sporadically causes mild disease in immunocompetent humans and Prospect Hill virus (PHV) so far has not been associated with any symptoms. They are thus defined here as low pathogenic/apathogenic hantavirus species. We found that productive infection of cells of the mononuclear phagocyte system (MPS), such as monocytes and dendritic cells (DCs), correlated well with the pathogenicity of hantavirus species tested. HTNV (intermediate case fatality rates) replicated more efficiently than PUUV (low case fatality rates) in myeloid cells, whereas low pathogenic/apathogenic hantavirus species did not produce any detectable virus titers. Analysis of PHPUV, a reassortant hantavirus derived from a pathogenic (PUUV) and an apathogenic (PHV) hantavirus species, indicated that the viral glycoproteins are not decisive for replication in MPS cells. Moreover, blocking acidification of endosomes with chloroquine decreased the number of TULV genomes in myeloid cells suggesting a post-entry block for low pathogenic/apathogenic hantavirus species in myeloid cells. Intriguingly, pathogenic but not low pathogenic/apathogenic hantavirus species induced conversion of monocytes into inflammatory DCs. The proinflammatory programming of MPS cells by pathogenic hantavirus species required integrin signaling and viral replication. Our findings indicate that the capacity to replicate in MPS cells is a prominent feature of hantaviral pathogenicity.

## Introduction

Hantaviruses (order *Bunyavirales*, family *Hantaviridae*) are globally emerging zoonotic pathogens that cause different types of viral hemorrhagic fever (VHF) (Kruger et al., 2015; Papa et al., 2016). The hallmarks of hantavirus-induced disease are increased vascular permeability as well as loss and dysfunction of platelets (Rasmuson et al., 2011; Vaheri et al., 2013b). In severe cases fatal shock and multiorgan failure can occur. The severity and case fatality rate of hantavirus-associated VHF depends on the hantavirus species involved (Jonsson et al., 2010; Kruger et al., 2011). Inadequate immune responses contribute to hantaviral pathogenesis (Schonrich et al., 2008, 2015; Schonrich and Raftery, 2016; Klingstrom et al., 2019).

Hantavirus species circulating in Europe and Asia, such as Puumala virus (PUUV), Hantaan virus (HTNV), and Dobrava-Belgrade virus (DOBV) are associated with hemorrhagic fever with renal syndrome (HFRS) with case fatality rates of <1% (PUUV), ~5% (HTNV), and ~10% (DOBV) (Vaheri et al., 2013a). Hantavirus species circulating in the Americas, such as Sin Nombre virus (SNV) and Andes virus (ANDV) can induce hantavirus cardiopulmonary syndrome (HCPS), a more severe form of VHF with case fatality rates of up to 40% (Macneil et al., 2011; Figueiredo et al., 2014). In contrast, hantavirus species that only sporadically cause mild disease, such as Tula virus (TULV) (Klempa et al., 2003; Zelena et al., 2013; Reynes et al., 2015) or infect humans without symptoms, such as Prospect Hill virus (PHV) (Yanagihara et al., 1984) are regarded as low pathogenic/apathogenic hantavirus species.

A group of myeloid cells that constitute the mononuclear phagocyte system (MPS) play a critical role in infectious diseases (Lugo-Villarino et al., 2019). The MPS cells—monocytes, macrophages and myeloid dendritic cells (DCs)—are distinct but morphologically and functionally similar professional antigen-presenting cells (APCs) that express major histocompatibility complex (MHC) class II molecules (Hume, 2006; Guilliams et al., 2014). They not only act as early checkpoints of the antiviral defense and stimulators of adaptive immunity but also substantially contribute to immunopathogenesis in zoonotic diseases caused by viruses, such as ebola virus, zika virus, dengue virus, and chikungunya virus (Supramaniam et al., 2018). The function of MPS cells is regulated by β2 integrins (Schittenhelm et al., 2017), which also serve as hantavirus receptors (Raftery et al., 2014). This suggests that hantaviral pathogenicity is linked to the MPS and β2 integrins. In accordance, several pathogenic hantavirus species (HTNV, PUUV, and ANDV) have previously been demonstrated to productively infect human DCs *in vitro* (Raftery et al., 2002a; Marsac et al., 2011; Scholz et al., 2017; Schonrich and Raftery, 2019). Previous studies also reported *in vitro* infection of primary monocytes (Nagai et al., 1985; Temonen et al., 1993; Scholz et al., 2017) or a monocytic cell line (Markotic et al., 2007) with pathogenic hantavirus species. It is unclear, however, whether the capacity to replicate in MPS cells differentiates pathogenic from low pathogenic/apathogenic hantavirus species.

In this study, we comparatively analyzed the capacity of pathogenic and low pathogenic/apathogenic hantavirus species to infect MPS cells. Moreover, we investigated the functional role of integrin signaling during hantavirus infection of MPS cells.

## Materials and Methods

### Vero E6 Cells

Vero E6 cells were cultured in Dulbecco's MEM (Gibco) supplemented with 10% hiFCS (BioWhittaker), 2 mM L-glutamine, penicillin, and streptomycin (PAA). Cells were passaged by washing with PBS (Biochrom), addition of trypsin until cells detached, and finally addition of FCS-containing medium to stop trypsin activity.

### Peripheral Blood Mononuclear Cell (PBMCs)

Density gradient centrifugation was used to isolate PBMCs from buffy coat units supplied by the German Red Cross (Dresden) as previously described (Raftery et al., 2002b). The PBMCs were derived from both female and male healthy donors. In brief, blood was diluted 1:1 with RPMI medium (RPMI 1640 with 2% heat-inactivated FCS, 0.2 mM EDTA) and placed on top of Ficoll-Hypaque (PAA). Tubes were subsequently centrifuged at 800 *g* for 30 min at room temperature. PBMCs were collected and washed twice with RPMI medium before use. PBMCs were cultured in RPMI 1640 supplemented with 10% hiFCS (BioWhittaker), 2 mM L-glutamine, penicillin, and streptomycin (PAA).

### Untouched Monocytes

Monocyte isolation kit II (Miltenyi Biotec) was used for isolation of untouched monocytes according to manufacturer's protocol. Subsequently, monocytes were cultured in RPMI 1640 supplemented with 10% hiFCS (BioWhittaker), 2 mM L-glutamine, penicillin and streptomycin (PAA).

### Isolation of Immature DCs (iDCs) From Peripheral Blood and Generation of Monocyte-Derived iDCs *in vitro*

Blood DC isolation kit II (Miltenyi Biotec) was used for isolation of myeloid iDCs from human PBMCs. Isolated iDCs were cultured in RPMI 1640 supplemented with 10% hiFCS (BioWhittaker), 2 mM L-glutamine, penicillin and streptomycin (PAA). Monocytes for *in vitro* generation of iDCs were isolated with CD14^+^ microbeads (Miltenyi Biotec). Subsequently, iDCs were generated from monocytes by adding a cytokine cocktail consisting of 500 IU/ml GM-CSF (ImmunoTools) and 200 IU/ml IL-4 (ImmunoTools). Medium and cytokines were changed every 2–3 days. Monocytes were also isolated from a LADIII patient (male) to generate LADIII iDCs *in vitro* as described above. The LADIII patient expressed normal levels of integrins but was defective in integrin signaling due to a homozygous mutation in *Fermitin Family Member 3* (*FERMT3*), which encodes kindlin-3, located in exon 12 (c.1525C>T) resulting in a premature stop codon (p.Arg509X) (Kuijpers et al., 2009).

### Humanized Mouse Model

Generation of mice with a humanized immune system has previously been published (Kobak et al., 2015). Briefly, NSG mice expressing HLA-A2, a human MHC class I molecule, were humanized by reconstitution with HLA-A2^+^ human CD34^+^ hematopoietic stem cells isolated from umbilical cord blood. Engraftment was evaluated at 11 weeks post-inoculation by cytofluorimetric analysis of PBMCs. Successfully engrafted mice were infected intraperitoneally (i.p.) with 10^5^ focus-forming units (FFU) of HTNV (strain 76-118). Infection was successful as determined by RT-qPCR from sera.

### Flow Cytometry

For surface staining, cells were harvested and washed twice in ice-cold FACS washing solution. Thereafter, cells were resuspended in 50 μl FACS blocking solution, containing the primary antibody in appropriate dilution (see below), and incubated for 1 h. After incubation, cells were again washed twice with FACS wash solution and for unconjugated primary antibodies, a corresponding labeled secondary antibody, diluted in FACS block solution, was added. After 45 min the cells were washed with FACS wash solution and resuspended in FACS fixation solution. For quantifying fluorescence of labeled cells a FACSCalibur® (BD Biosciences) was used. Results were evaluated with the flow cytometry analysis software program CellQuestPro® (BD Biosciences) and FlowJo™ Software (BD Biosciences).

### Viruses and Infection

Virus stocks of HTNV (strain 76-118), TULV (strain Lodz), PHV (strain 3571), PHPUV (Handke et al., 2010), DOBV (genotype Sochi), SNV, and K26GFP were propagated on VeroE6 cells. K26GFP was derived from HSV-1 strain KOS and expresses green fluorescent protein (GFP) coupled to the 12 kDa capsid protein designated VP26, which is encoded by open reading frame UL35 (Desai and Person, 1998). Hantaviruses were propagated and titrated in a biosafety level 3 (BSL3) laboratory as previously described (Kraus et al., 2005). Virus stocks were regularly tested for mycoplasma contamination by PCR and stored at −80°C before use. In order to infect cells, virions were allowed to adsorb to cells for 1 h at 37°C. Thereafter, cells were washed three times with medium before being seeded in a cell culture vessel at a density of 10^6^/ml and incubated in a humidified atmosphere at 37°C. Uninfected cells treated with medium instead of virions were used as mock control.

### Reagents, TLR Ligands, and Cytokines

In order to block integrin signaling the Src kinase inhibitor PP2 (Merck) at 10 μM in DMSO was used. In addition, the following TLR ligands, cytokines, and reagents were used: LPS (InvivoGen) at 1 μg/ml, poly I:C (Sigma) at 1 μg/ml, IFNα-2a (ImmunoTools) at 5,000 U/ml, chloroquine (Sigma) at 10 μM, and phorbol 12-myristate 13-acetate (PMA) (Sigma) at 10 nM.

### Antibodies and Staining Reagents

Anti-CD14 (clone M5E2), anti-CD83 (clone HB15e), anti-CD107a (clone H4A3), and anti-integrin αM (clone 44), were supplied by BD PharMingen; anti-integrin β1 (clone MEM-101), anti-integrin β3 (clone C17), and anti-CD86 (clone IT2.2) were supplied by ImmunoTools; anti-DC-SIGN (Clone MR-1) was purchased from Acris; anti-gC1qR (clone 74.5.2) was supplied from Chemicon; anti-CD55/DAF (clone 143-30) was purchased from Southern Biotechnology; anti-CD51 (anti-integrin αv) (clone 13C2) was supplied from QED Science. Anti-MHC class II (clone L243) was produced in-house. Annexin V-FITC (ImmunoTools) was used to detect phosphatidyl serine residues on the surface of apoptotic cells. TUNEL assay was performed using *In situ* TUNEL kit (Roche). Hantavirus nucleocapsid (N) protein was stained with N-specific polyclonal rabbit serum (Razanskiene et al., 2004), monoclonal antibody 1C12 (Lundkvist et al., 1991) or pig anti-hantavirus N protein sera. The pig antisera were raised against yeast-expressed N proteins of DOBV, PUUV or TULV according to a standard protocol. The anti-DOBV N protein polyclonal pig serum cross-reacted with HTNV N protein. Anti-ß-actin (clone ab6276) was purchased from Abcam. Polyclonal rabbit anti-human B2M (hB2M) was supplied by Dako. Anti-MxA monoclonal antibody M143 was kindly supplied by O. Haller. Anti-MHC-I heavy chain D226-3 was supplied by Biozol, Eching. Isotype-matched control antibodies were supplied by BD Pharmingen. Secondary antibodies coupled to fluorochromes or horseradish peroxidase were supplied by Dianova.

### Immunohistochemistry and Immunocytometry

Infected DCs were adhered to poly-L-lysine treated slides for 10 min before being fixed with cold 1% paraformaldehyde in PBS for 20 min at 4°C. Cells were then stained for hantavirus N protein and MHC class II as previously described (Kraus et al., 2005). Fluorescence microscopy was performed on an Olympus BX60 microscope, confocal analysis on a Leica DM 2500 and LCS software. Primary and secondary antibodies were used at a 1:300 dilution for immunocytometry, whereas a 1:100 dilution was used for immunohistochemistry of formalin-fixed paraffin-embedded sections. Deparaffination was perfomed by standard procedures and autofluorescence was quenched using a Sudan B 0.1% solution.

### Western Blot

For infection, iDCs and bDCs were incubated with live virus using a multiplicity of infection (MOI) of 1 or 1.5 for 1 h at 37°C. The cells were then washed three times with RPMI 1640 containing 5% heat-inactivated FCS before being resuspended in the appropriate medium at a density of 10^6^/ml. After incubation as indicated in the figure legends, cells were lysed in lysis buffer (250 mM Tris, 2% SDS, 10% glycerol, 5% mercaptoethanol, 0.01% bromphenol blue) followed by heat-denaturation of the sample for 5 min at 95°C. Proteins were separated by 10% sodium dodecyl sulfate (SDS)-polyacrylamide gel electrophoresis and transferred onto a PVDF membrane (Millipore) before blocking and staining for hantavirus N protein and β-actin.

### RT-qPCR

Light cycler RT-qPCR has been previously described (Lutteke et al., 2010). Briefly, cells were lysed with MagNA Pure lysis buffer (Roche) and mRNA was isolated with a MagNA Pure-LC device. Subsequently, RNA was reverse-transcribed with avian myeloblastosis virus reverse transcriptase (AMV-RT) and oligo (dT) primer using the First Strand cDNA Synthesis Kit from Roche. Special LightCycler Primer Sets (Search-LC) were used with LightCycler FastStart DNA Sybr Green I Kit (Roche) in order to amplify targets. The input RNA was normalized using average expression of β*-actin* and *cyclophilin B* housekeeping genes. In order to generate a virtual standard curve a known input concentration of a plasmid was plotted to the PCR cycle number at which the fluorescence intensity reached a fixed value. The standard curve was used to calculate transcript copy numbers. The relative copy numbers represent mean averages of data from two independent analyses for each sample and parameter. For RT-qPCR of hantavirus genomes primer sets binding to a highly conserved region within the S-segment of hantavirus genomes were used as previously published (Kramski et al., 2007).

### Heat Maps

For generation of heat maps, mean values from the RT-qPCR analysis of iDCs derived from three different donors were normalized to the maximum positive control value for each gene giving values from 0 (no expression; light blue) to 1 (maximum expression; red).

### Statistical Analysis

Student's *t*-test was used to determine statistical significance. *P*-values below 0.05 (95% confidence) were considered to be significant. Prism 5 software (GraphPad) was used for statistical analysis.

## Results

### Susceptibility of MPS Cells to HTNV but Not TULV

**Figure 1:**
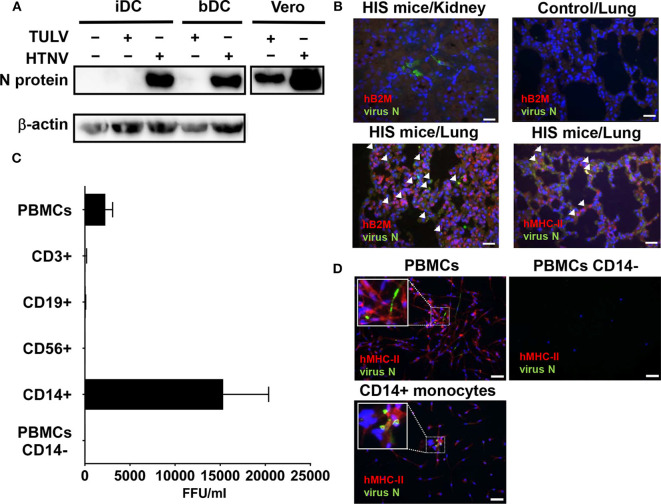
Susceptibility of MPS cells to HTNV but not TULV. **(A)** Detection of hantavirus N protein in monocyte-derived iDCs or iDCs isolated from blood (bDCs) at 5 days p.i. with HTNV or TULV (MOI 1). As a positive control, Vero E6 cells were also infected with HTNV or TULV. Results shown are representative for three independent experiments. **(B)** Humanized immune system (HIS) mice or unreconstituted mice (Control) were infected for 22 days before sacrifice, fixation and embedding of lung or kidney tissues in paraffin. Slices were prepared and stained with anti-DOBV N protein polyclonal pig serum, which cross-reacts with HTNV N protein (FITC, green), DNA (DAPI, blue) and either polyclonal rabbit anti-human B2M (Alexa 594, red) or anti-human MHC-II (Alexa 594, red). Arrows indicate the presence of infected human cells. Scale bar is 100 μm. **(C)** PBMCs, PBMCs depleted of CD14^+^ monocytes (PBMCs CD14^−^) and PBMC subsets (CD14^+^ monocytes, CD3^+^ T cells, CD19^+^ B cells, and CD56^+^ NK cells) were purified by density gradient and MACS from healthy human donors and infected with HTNV (MOI of 0.1). After 6 days incubation supernatant was assessed for viral replication. Viral titers are given as focus-forming units per ml (FFU/ml). Results are derived from 3 to 5 independent experiments. **(D)** Cells infected as for **(C)** were stained using anti-DOBV N protein polyclonal pig serum, which cross-reacts with HTNV N protein (FITC, green), monoclonal anti-human MHC-II (Alexa 594, red) and DNA (DAPI, blue). Inserts show three times magnified areas with characteristic HTNV N staining in PBMCs and CD14^+^ monocytes but not in PBMCs depleted of CD14^+^ cells (PBMCs CD14^−^). Scale bar is 100 μm.

First, we comparatively studied the ability of pathogenic (HTNV) and low pathogenic (TULV) hantavirus species to infect monocyte-derived immature DCs (iDCs) or iDCs isolated from peripheral blood (bDCs). At 5 days post-infection (p.i.), hantavirus N protein was detected in all HTNV-infected cells but neither in iDCs nor in bDCs infected with TULV (Figure 1A). In contrast, viral N protein was found in both HTNV-infected and TULV-infected Vero E6 cells (Figure 1A). Vero E6 cells are of epithelial origin and lack type I interferon (IFN) genes (Diaz et al., 1988). This result indicates that MPS cells, such as iDCs are susceptible to HTNV but not TULV.

Next we investigated HTNV infection of MPS cells in mice with a humanized immune system (HIS), a valuable tool in VHF research (Schonrich and Raftery, 2017). In fact, renal tissue from HTNV-infected HIS mice showed few infiltrating human immune cells and only localized areas of infection (Figure 1B, top left). Lung tissue from HTNV-infected HIS mice, however, showed widespread infection and infiltrating human immune cells that were identified by expression of human β2 microglobulin (Figure 1B, bottom left) and human MHC class II molecules (Figure 1B, bottom right).

The human MHC-II^+^ cells in HIS mice could have been human MPS cells or human lymphocytes (B cells and T cells) which can also express MHC class II molecules (Costantino et al., 2012). To differentiate between these possibilities, we investigated the infectability of human peripheral blood mononuclear cells (PBMCs) that mainly consist of lymphocytes (in the order of frequency: T cells, B cells, NK cells) and monocytes (3–10% of peripheral blood cells). Indeed, PBMCs derived from healthy human donors produced significant viral titers after 6 days incubation with HTNV *in vitro* (Figure 1C). In contrast, PBMCs inoculated with TULV showed no obvious viral replication at 6 days after inoculation (data not shown). In order to pinpoint the cell type most susceptible to HTNV, common lymphocyte subsets (CD3^+^ T cells, CD19^+^ B cells, CD56^+^ NK cells) and common myeloid cells (CD14^+^ monocytes) were isolated by magnetic cell sorting (MACS) and subsequently inoculated with HTNV. Of these, monocytes but not lymphocytes generated significant HTNV titers (Figure 1C). Confirming this result, PBMCs depleted of CD14^+^ monocytes (PBMCs CD14^−^) and infected with HTNV did not produce significant viral titers (Figure 1C). In addition, immunofluorescence analysis of HTNV-infected PBMCs showed infection of MHC class II expressing adherent cells, whereas PBMCs depleted of CD14^+^ monocytes (PBMCs CD14^−^) had both fewer adherent cells and no sign of infection (Figure 1D, upper panel). HTNV-Infected CD14^+^ monocytes, however, showed hantaviral N protein in patterns of small threads and inclusions typical of productive hantavirus infection (Figure 1D, lower panel). TULV infected cells showed no significant staining (data not given).

Taken together, cells of the MPS system support replication of HTNV but not TULV.

### Association of Hantavirus Pathogenicity and Hantavirus Replication in MPS Cells

**Figure 2:**
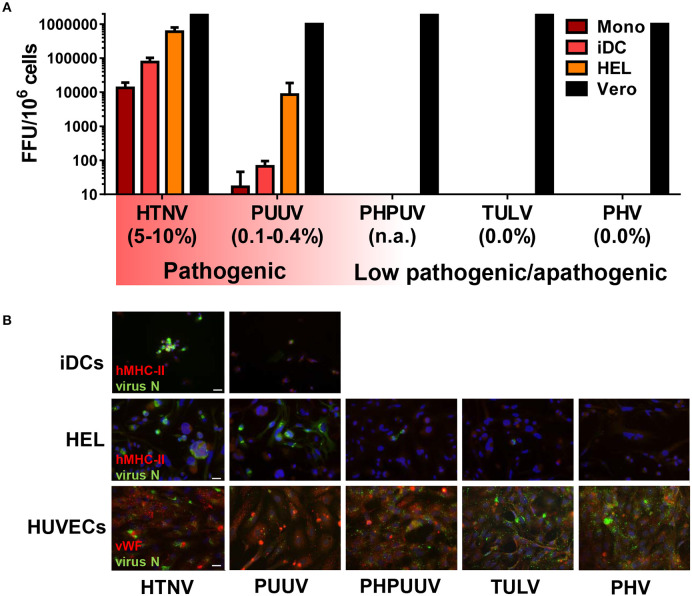
Relationship between hantavirus pathogenicity and hantavirus replication in the MPS. **(A)** Titration assay of virus released from infected myeloid cells. CD14^+^ monocytes (Mono), CD14^+^ monocytes stimulated with GM-CSF and IL-4 (iDCs), human megakaryocyte cell line HEL stimulated with 10 nM PMA (HEL), and Vero E6 cells (Vero) were infected with hantaviruses (MOI of 1) for 4 days. Viral titers are given as focus-forming units (FFU), the reported case fatality rates are given in brackets (Jonsson et al., 2010; Vaheri et al., 2013a). **(B)** Cells were infected as for **(A)** except that virus stocks were filtered by a 0.2 μm filter to remove possible debris that might be uptaken by phagocytosis, and that HUVECs were used instead of Vero E6 cells. After 4 days cells were fixed, stained for hantavirus N protein (FITC, green), DNA (DAPI, blue), and for either MHC class II (Texas red, red) (Top and middle row) or the endothelial marker von Willebrand factor (vWF) (Texas red, red) (Bottom row) before immunofluorescence microscopy. Scale bar is 20 μm.

Next, we extended the spectrum of hantavirus species with distinct pathogenic potential in our analysis. Besides HTNV and TULV we included PHV, which can infect humans but has never been associated with human disease (Yanagihara et al., 1984), and PUUV, which unlike HTNV only causes mild to moderate HFRS that is rarely fatal (Vaheri et al., 2013a). We also analyzed PHPUV, a reassortant hantavirus virus. The hantaviral RNA genome consists of a small (S), medium (M), and large (L) segment (Elliott and Schmaljohn, 2014). The L- and S-segment of PHPUV are derived from PHV, whereas the M-segment encoding the envelope glycoproteins originates from PUUV (Handke et al., 2010). These different hantavirus species were used to infect CD14^+^ monocytes, iDCs derived from CD14^+^ monocytes, and HEL cells, a promegakaryocyte cell line of myeloid origin similar to MPS cells, which support HTNV replication (Lutteke et al., 2010). We found that HTNV replicated more efficiently than PUUV (low case fatality rate) in myeloid cells, whereas low pathogenic (TULV) and apathogenic (PHV) hantavirus species did not produce any detectable virus titers (Figure 2A). Intriguingly, the reassortant hantavirus PHPUV did not replicate, although it expressed the envelope glycoproteins from a pathogenic hantavirus (Figure 2A). In stark contrast, all hantavirus species and PHPUV could infect Vero E6 cells (Figure 2A).

To confirm these findings we examined hantavirus N protein expression in human myeloid cells (iDCs, HEL cells) and for comparison in human umbilical vein endothelial cells (HUVECs). Immunofluorescence analysis at 4 days p.i. (MOI of 1) revealed strong N protein expression in HTNV-infected iDCs but less in PUUV-infected iDCs (Figure 2B, upper row). No iDCs were found positive for PHV, PHPUV, or TULV (data not shown). HEL cells were permissive to HTNV and PUUV but only sporadically showed expression of hantavirus N protein after exposure to PHV, PHPUV, or TULV (Figure 2B, middle row), suggesting viral entry was taking place but replication was inhibited. As expected, HUVECs were susceptible to infection with all hantavirus species tested, albeit to a different extent (Figure 2B, bottom row). Similar to other pathogenic hantaviruses, DOBV and SNV could also infect iDCs and HEL cells (Figure S1).

These results together suggest that the capacity to replicate in myeloid cells differentiates pathogenic from low pathogenic/apathogenic hantavirus species.

### Evidence for Post-entry Block of TULV Replication in the MPS

**Figure 3:**
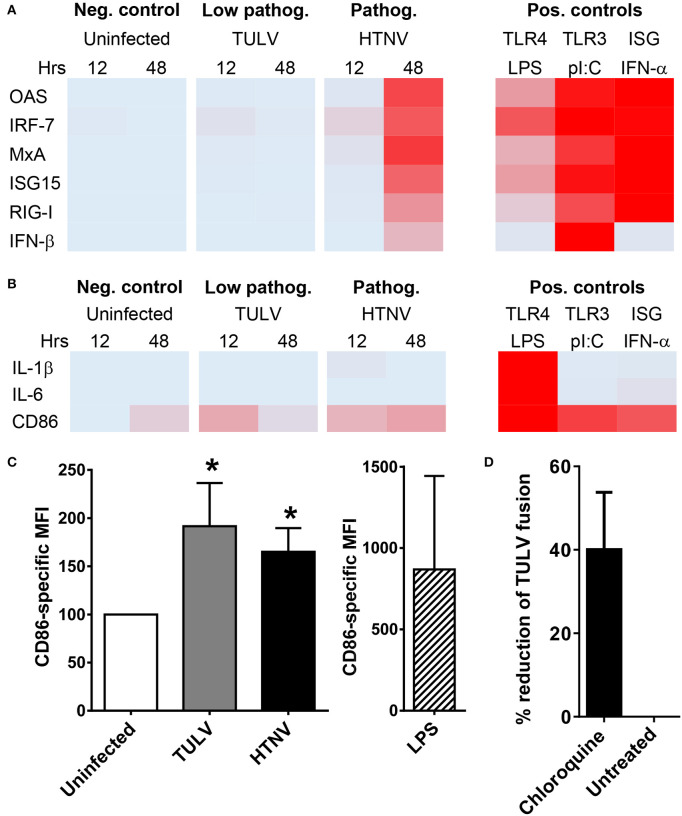
Post-entry block of TULV replication in myeloid cells. **(A)** iDCs were left uninfected or infected with either HTNV or TULV (MOI of 1.5) for 12 or 48 h. As positive controls cells were treated with LPS (1 μg/ml), poly I:C (1 μg/ml), or IFN-α 2a (5,000 U/ml) for 8 h. Heat map of RT-qPCR analysis of **(A)** ISGs (OAS, IRF-7, MxA, ISG15, RIG-I, and IFN-β) and of **(B)** NF-kB driven genes (IL-1β, IL-6, CD86). The RT-qPCR analyses corresponding to the heat maps are shown in Figures S4, S5. Mean values from three different donors were normalized to the maximum positive control value for each gene giving values from 0 (no expression; light blue) to 1 (maximum expression; red). **(C)** Flow cytometric analysis of CD86 expression on iDCs infected for 12 h. The data are shown as CD86-specific MFI. LPS-stimulated iDCs served as a maximum control. Error bars represent the mean ± SD. The data are derived from iDCs from three different donors (*p < 0.05, Students *t*-test). **(D)** HEL cells were pretreated with 2 mM chloroquine or left untreated before being incubated with TULV (MOI of 5) for 2 h at 37°C. Cells were washed thoroughly with PBS with 50 μg/ml heparin before being harvested and analyzed by RT-qPCR for TULV genome. The reduction in viral genome numbers relative to untreated cells is shown, error bar represents mean ± SEM of four independent experiments.

Now we explored why low pathogenic/apathogeneic hantavirus species do not replicate in myeloid cells. Hantavirus replication is known to trigger endoplasmic and cytoplasmic pattern recognition receptors (PRR), i.e., toll-like receptor (TLR)3 (Handke et al., 2009) and retinoic acid inducible gene I (RIG-I) (Lee et al., 2011). The resulting signaling cascades induce mostly IFN-β and IFN-stimulated genes (ISGs) that interfere with viral replication. Intriguingly, induction of ISGs in TULV-infected iDCs was barely detectable and did not increase with time (Figure 3A). In contrast, HTNV clearly induced IFN-β and ISGs although mostly later in infection (Figure 3A). Both hantavirus species failed to induce NF-kB driven transcripts, such as IL-6 and IL-1β within 48 h of infection (Figure 3B). In contrast, both HTNV and TULV increased expression of transcripts encoding CD86, a key T cell costimulatory molecule (Figure 3B). The TULV-induced effect on CD86 expression, however, was short-lived and no longer visible at 48 p.i., whereas, the CD86 transcript level of HTNV-infected iDCs remained elevated at this time point (Figure 3B). Flow cytometric analysis confirmed the upregulation of CD86 molecules on iDCs exposed to either HTNV or TULV for 12 h (Figure 3C). In this type of experiment, LPS-stimulated iDCs served as a maximum control. These data exclude that an overwhelming cell-intrinsic immune response abrogates TULV replication in iDCs.

MPS cells strongly express integrin β1, the only receptor described for low pathogenic/apathogenic hantavirus species (Gavrilovskaya et al., 1998, 1999) as well as receptors described for pathogenic hantavirus species (integrin β2, integrin β3, gC1qR, and CD55) (Figure S2) (Gavrilovskaya et al., 1998, 1999; Choi et al., 2008; Krautkramer and Zeier, 2008; Raftery et al., 2014). After receptor binding hantaviruses enter target cells by an endocytic pathway and are finally released from endosomes into the cytoplasm (Albornoz et al., 2016; Mittler et al., 2019). The latter process requires acidification of endosomes, which can be blocked by lysosomotropic bases, such as chloroquine (Mercer et al., 2010). We found that pre-treatment with chloroquine before incubation with TULV reduced the number of viral genomes detected in HEL cells suggesting that TULV can enter myeloid cells (Figure 3D).

Taken together, these results are consistent with the idea that replication of low pathogenic/apathogenic hantavirus species in MPS cells is blocked early after entry thereby preventing induction of a vigorous cell-intrinsic immune response.

### Requirement of Integrin Signaling for Proinflammatory Programming of MPS Cells by Hantaviruses

**Figure 4:**
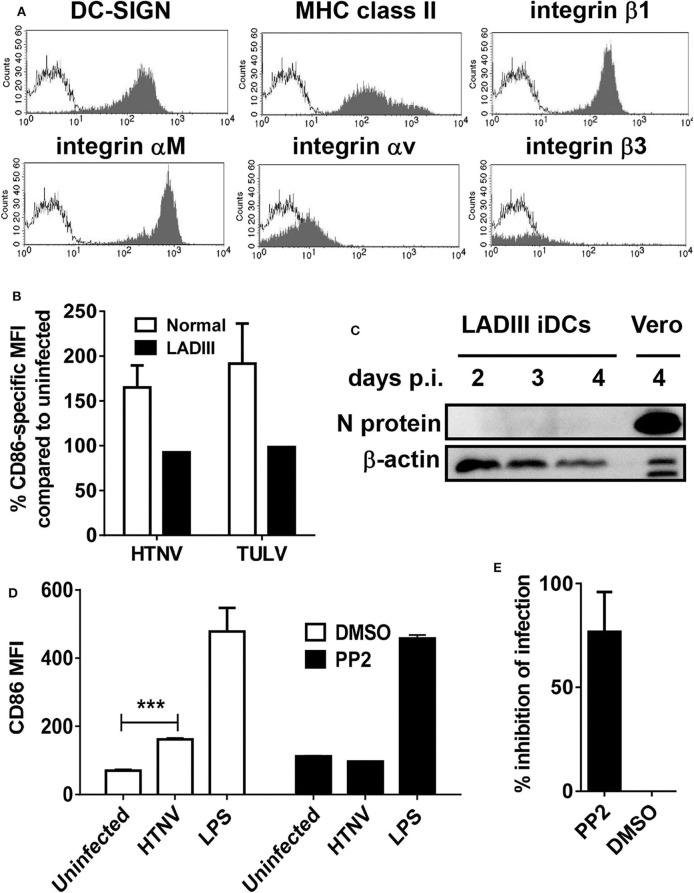
Hantavirus-induced DC maturation requires integrin signaling. **(A)** LADIII iDCs were stained for DC-SIGN, MHC class II molecules, integrin β1, integrin αM, integrin αv, and integrin β3 before flow cytometric analysis. The x-axis shows fluorescence intensity (log scale), and the y-axis depicts the relative cell number (open curve: isotype control). **(B)** LADIII iDCs and normal iDCs were left uninfected or infected with either HTNV or TULV (MOI of 1.5) for 12 h. Subsequently, cells were stained for CD86 and analyzed by flow cytometry. The CD86 expression on infected cells is given as a percentage of CD86 expression on the corresponding uninfected cells. Results shown are representative of three experiments. **(C)** LADIII iDCs were infected with HTNV (MOI of 1.5) for the time indicated, harvested and analyzed by western blot for expression of hantavirus N protein. **(D)** Normal iDC pretreated with PP2 (1 mM) or DMSO (1 μl/ml) for 30 min before being exposed to HTNV (MOI of 1.5) or treated with LPS (1 μg/ml) for 12 h. Subsequently, cells were stained for CD86 and analyzed by flow cytometry. Results shown are representative of three experiments (***p < 0.001; Students *t-*test). **(E)** HEL cells pretreated with PP2 (1 mM in DMSO) or DMSO (1 μl/ml) as a control were infected with HTNV (MOI of 0.5) for 1 h before being washed three times and incubated for 3 days. After staining for hantavirus N protein (FITC) and DNA (DAPI), cells were counted in 4 fields of view and infected cells were determined by N protein expression. The percentage of inhibition of infection in PP2-treated cells as compared to cells treated with DMSO is shown. Results are derived from three independent experiments; error bars represent mean ± SD.

We now tested whether integrin signaling is involved in the MPS maturation program driven by pathogenic hantavirus species. For this purpose we used iDCs from a patient with the extremely rare leukocyte adhesion deficiency (LAD)III, which shows impaired signaling through β1, β2, and β3 integrins (Kuijpers et al., 2009). LADIII iDCs were phenotypically normal (Figure 4A). Similar to normal iDCs, upon infection with herpes simplex virus type 1 (HSV-1) LADIII iDCs downregulated CD83 (Figure S3), a reaction that is proteasome-dependent and not known to require integrin signaling (Salio et al., 1999; Kruse et al., 2000; Kummer et al., 2007). In stark contrast to normal iDCs, however, LADIII iDCs did not upregulate the CD86 molecules in response to HTNV and TULV, respectively, at 12 h after infection (Figure 4B). Intriguingly, the hantaviral N protein was not detectable in HTNV-infected LADIII iDCs suggesting that HTNV replication requires integrin signaling (Figure 4C). We confirmed these findings by blocking hantavirus-induced upregulation of CD86 on healthy iDCs with PP2 (Figure 4D), which blocks Src tyrosine kinases that play a pivotal role in outside-in-signaling through β2 integrins (Berton et al., 1994). In contrast, integrin-independent CD86 upregulation through stimulation of TLR4 with LPS was not affected by PP2 (Figure 4D). Pretreatment with PP2 also inhibited HTNV infection in HEL cells (Figure 4E).

In conclusion, these data indicate that integrin signaling facilitates replication of pathogenic hantavirus species in myeloid cells and proinflammatory programming of the MPS.

### Induction of Inflammatory DCs by Hantavirus Replication in Monocytes

**Figure 5:**
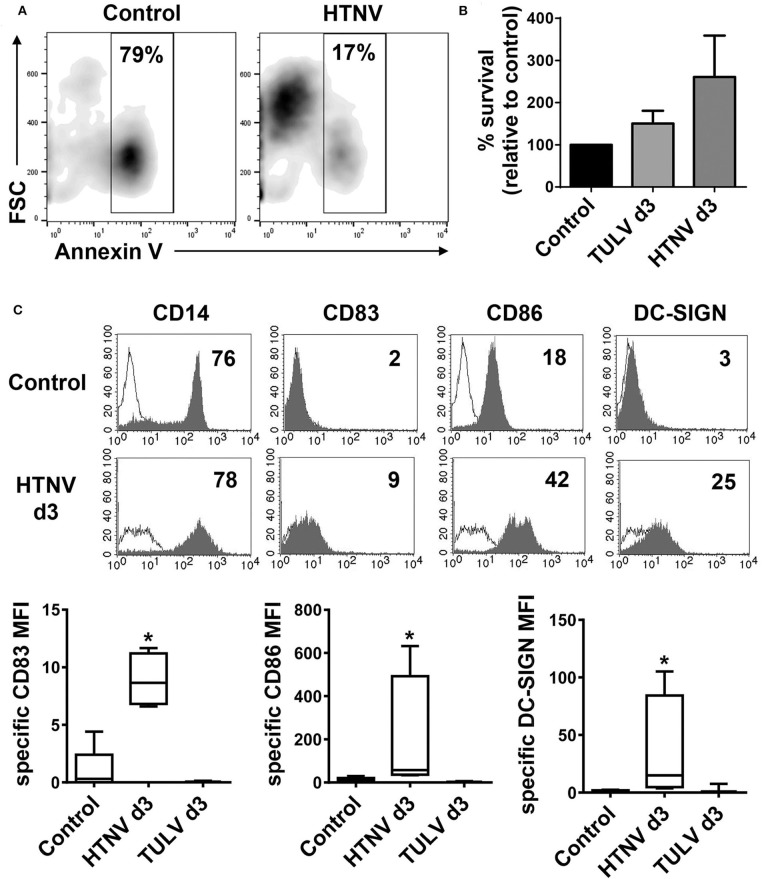
Generation of inflammatory DCs upon HTNV infection of monocytes. CD14^+^ monocytes derived from healthy human donors were left uninfected (Control) or infected with HTNV (MOI of 1). **(A)** Cells were stained with Annexin V-FITC before flow cytometric analysis. The x-axis depicts Annexin V-staining whereas the y-axis shows cell size according to forward Scatter (FSC). Percentage of Annexin V-positive cells is indicated in the analysis gate. Results from one out of two independent experiments are shown. **(B)** Uninfected cells (Control) or cells infected with TULV or HTNV (MOI of 1) for 3 days were stained with Annexin V (PE) and DNA fragmentation was labeled by TUNEL (FITC) staining. The percentage of surviving (PE-negative, FITC-negative) is given relative to uninfected cells (Control). The data are derived from three independent experiments, error bars represent mean ± SEM. **(C)** Monocytes were infected with HTNV or TULV and cultured for 3 days before being analyzed by flow cytometry for expression of CD14, CD83, CD86, and DC-SIGN. Uninfected monocytes on the day of isolation (d0) were used as a control as they rapidly undergo spontaneous apoptosis in culture. Upper panel: histograms showing one representative analysis of d3 HTNV-infected monocytes (open curves: isotype control). The x-axis shows staining with specific antibody as mean fluorescence intensity (MFI) whereas the y-axis indicates cell count. The relevant MFI is given in the upper right corner. Lower panel: Box-and-whisker plots of statistical analysis of DC-SIGN, CD83, and CD86 expression on uninfected monocytes (d0), HTNV-infected monocytes (d3), or TULV-infected monocytes (d3). The data are derived from at least three independent experiments (*p < 0.05; Students *t*-test).

Next, we investigated the functional impact of hantavirus replication in human CD14^+^ monocytes. These cells circulate in the peripheral blood before they enter tissue and differentiate during inflammation. In culture, however, human monocytes are short-lived and undergo spontaneous apoptosis in the absence of external survival signals, such as cytokines or microbial products (Mangan et al., 1991). In accordance, the majority of uninfected monocytes (Control) but only a relatively low percentage of HTNV-infected monocytes were Annexin V-positive after 3 days of culture (Figure 5A). In a further experiment, we stained uninfected (Control) and infected monocytes after 3 days of culture for both Annexin V (PE) and DNA fragmentation (FITC), which is a more stringent test for apoptosis. Subsequently, we calculated the percentage of surviving cells (PE-negative and FITC-negative) relative to the uninfected control (Figure 5B). There was a tendency toward more survival in HTNV-infected monocytes as compared to TULV-infected and uninfected cells although this difference was statistically not significant. Intriguingly, cytofluorimetric analysis revealed that HTNV-infected monocytes developed into inflammatory DCs. They showed upregulation of DC maturation marker CD83, the co-stimulatory molecule CD86, and the DC-specific C-type lectin ICAM-3-grabbing non-integrin (DC-SIGN) on day 3 of culture (Figure 5C, histograms in the lower row and box-and-whisker plots). In this type of analysis, uninfected monocytes on the day of isolation served as a control (Figure 5C, histograms in the upper row and box-and-whisker plots) due to their spontaneous apoptosis in culture. In contrast to HTNV, TULV did not induce upregulation of these markers on monocytes during culture (Figure 5C, box-and-whisker plots).

These results indicate that pathogenic but not low pathogenic/apathogenic hantavirus species induce the generation of inflammatory DCs that could contribute to both clearance of hantavirus-infected cells and hantavirus-associated immunopathology.

## Discussion

In this study, we provide evidence that hantavirus pathogenicity is associated with replication in MPS cells resulting in proinflammatory programming of the MPS. Our data indicate that the failure of low pathogenic/apathogenic hantavirus species to replicate in MPS cells is due to a post-entry block rather than an overwhelming innate response. Furthermore, our experimental results suggest that integrin signaling is required for proinflammatory programming of MPS cells by pathogenic hantavirus species.

Due to their much higher frequency in PBMCs, monocytes rather than DCs probably contribute the most to replication of pathogenic hantavirus species in peripheral blood. In contrast to monocytes, common lymphoid cell types (NK cells, T cells, B cells) were refractory to infection with pathogenic hantavirus, as we have previously shown for granulocytes (Raftery et al., 2014). We cannot exclude, however, that rare lymphoid cell subsets are hantavirus permissive. In monocytes, DCs and HEL cells, HTNV (intermediate pathogenicity) replicated more efficiently than PUUV (low pathogenicity) whereas low pathogenic/apathogenic hantavirus species, such as TULV and PHV did not generate significant virus titers. This observation is in accordance with a recent study that did not find TULV replication in human macrophages (Bourquain et al., 2019). Moreover, clinical studies of acute HFRS detected increased numbers of circulating monocytes and provided evidence for a positive correlation between monocyte activation and severity of HFRS (Wang et al., 2014; Tang et al., 2015; Li et al., 2018). Altogether, these findings strongly suggest that the capacity of hantavirus species to replicate in MPS cells determines their pathogenic potential.

The reassortant hantavirus PHPUV did not replicate in MPS cells although it expressed the envelope glycoproteins from a pathogenic hantavirus species. Moreover, TULV and PHV failed to productively infect MPS cells despite high levels of β1 integrin, the receptor for low pathogenic/apathogenic hantavirus species (Gavrilovskaya et al., 1998, 1999). Thus, hantaviral receptors and their ligands are not central for the differential MPS cell tropism of hantavirus species. An overwhelming cell-intrinsic immune response could have terminated the replication of low pathogenic/apathogenic hantavirus species after entry into myeloid cells as observed for endothelial cells (Geimonen et al., 2002; Kraus et al., 2004; Rang, 2010). This can be ruled out, however, as TULV did not activate significant expression of ISGs in iDCs. In addition, TULV only transiently upregulated CD86 on DCs, possibly due to binding to and entry via β1 or β2 integrins. Similarly, UV-inactivated HTNV did not induce sustained upregulation of CD86 on iDCs (Raftery et al., 2002a). In stark contrast, replication-competent HTNV induced a clear and long lasting proinflammatory signature in myeloid cells. This suggests that proinflammatory programming of MPS cells by pathogenic hantavirus species requires both outside-in-signaling through integrins and signals emanating from cell-intrinsic sensors of viral replication, such as RIG-I.

We detected reduced numbers of TULV genomes at 2 h post-infection in myeloid cells pretreated with chloroquine as compared to untreated control cells. This is not due to viral replication in untreated cells as the copy number of viral RNAs does not start to increase before 9 h post-infection with Old World hantavirus species (MOI of 10) (Wigren Bystrom et al., 2018). Importantly, no increase is detected at 2, 5, and 7 h post-infection (Wigren Bystrom et al., 2018). Old world hantavirus species enter cells via clathrin-coated pits (Jin et al., 2002). Chloroquine not only blocks acidification of endosomes and release of viral particles from endosomes into the cytoplasm but also reduces the rate of endocytosis via clathrin coated pits (Hu et al., 2020). The latter is due to inhibitory effect of chloroquine on expression of phosphatidylinositol binding clathrin assembly protein (PICALM), which represents one of the three most abundant proteins in clathrin-coated pits (Wolfram et al., 2017). Altogether, these findings suggest that low pathogenic/apathogenic hantavirus species, such as TULV can enter myeloid cells via clathrin-coated pits but do not replicate due to an not yet defined post-entry block.

We observed that CD14^+^ monocytes infected with HTNV *in vitro* differentiate into inflammatory DCs with upregulation of functionally important molecules, such as C-type lectin DC-SIGN, DC maturation marker CD83 and the T cell-stimulatory molecule CD86. In accordance, it has been reported that HTNV-infected monocytes acquire the morphology of DC-like cells (Markotic et al., 2007) and PUUV-infected monocytes increase expression of CD86 (Scholz et al., 2017). For other viruses (influenza A virus, vesicular stomatitis virus, vaccinia virus) it has been demonstrated that virus-induced differentiation of CD14^+^ monocytes into inflammatory DCs is independent of cell division, requires viral gene expression, and is not associated with induction of cell death (Hou et al., 2012). In line with these findings, we observed a tendency toward more survival in HTNV-infected monocyte cultures as compared to uninfected control cultures after 3 days of culture. Similarly, PUUV-infected monocytes showed increased survival compared to uninfected control cells (Scholz et al., 2017). It is likely that autocrine GM-CSF and tumor necrosis factor (TNF)-α promote HNTV-induced monocyte differentiation into DCs as suggested for other viruses (Hou et al., 2012). Type I IFN also promotes the differentiation of monocytes into DCs (Blanco et al., 2001).

During inflammatory processes monocytes leave the bloodstream to enter the tissue and develop into inflammatory DCs that substantially contribute to virus elimination and also cause immunopathogenesis (Shi and Pamer, 2011). A significant influx of mononuclear cells expressing MHC class II molecules is detected in mucosal biopsies from the airways of patients during the acute stage of mild HFRS (Scholz et al., 2017). In accordance, by analyzing a mouse model of hantavirus infection based on HIS mice we detected hantaviral N protein in lung cells expressing human MHC class II molecules. In this mouse model, the highest numbers of viral genomes are found in the lung (Kobak et al., 2015). This results in non-cardiogenic acute pulmonary edema due to capillary leakage, which is observed in both HFRS and HPS (Clement et al., 2014), whereas kidney function is not affected (Kobak et al., 2015). The lung infiltrating cells were professional human APCs as human lymphocytes, the only cells that also can express functional MHC class II molecules (Costantino et al., 2012), are not susceptible to hantavirus infection.

It is likely that during infection with pathogenic hantavirus species monocytes mobilized from the peripheral blood enter the lung and further differentiate into inflammatory DCs, which are as active as classical DCs with regard to antigen-presenting function (Cheong et al., 2010). The generation of inflammatory DCs may significantly contribute to activation of virus-specific CD8^+^ T cells during the acute phase of HFRS. Moreover, hantavirus-infected CD14^+^ monocytes induce bystander T cell activation (Raftery et al., 2018). Thus, hantavirus-driven monocyte-to-DC conversion may contribute to both virus elimination and virus- immunopathology.

We collected further evidence that integrin signaling is important for hantavirus-driven differentiation of MPS cells. Firstly, iDCs derived from a LADIII patient showed a normal phenotype but failed to upregulate CD86 in response to hantavirus due to defective integrin signaling. Secondly, normal iDCs did not increase CD86 expression upon HTNV infection in the presence of PP2, which efficiently blocks outside-in-signaling through β2 integrins (Berton et al., 1994). Thirdly, hantavirus N protein was not detectable in HTNV-infected LADIII iDCs and hantavirus infection of normal iDCs was inhibited by pretreatment with PP2. These observations imply that intact integrin signaling is required for hantavirus-induced generation of inflammatory DCs. In line with this view, monocyte-to-DC conversion in a model of transendothelial trafficking was dependent on β2 integrins (Randolph et al., 1998).

In conclusion, hantavirus replication in MPS cells drives differentiation of these important immune cells and is a feature that differentiates pathogenic from low pathogenic/apathogenic hantavirus species. The correlation between hantaviral pathogenicity and replication in MPS cells could be exploited to evaluate the considerable number of hantavirus species with unknown pathogenicity in humans.

## Data Availability Statement

All datasets generated for this study are included in the article/Supplementary Material.

## Ethics Statement

The studies involving human participants were reviewed and approved by Ethics committe of the Charité–Universitätsmedizin Berlin. The patients/participants provided their written informed consent to participate in this study. The animal study was reviewed and approved by Landesamt für Gesundheit und Soziales Berlin G 0013/12.

## Author Contributions

MR, PL, GS, LK, LR, and TG conceived and designed the experiments. MR, PL, LK, LR, and NL performed the experiments. MR, PL, GS, LR, and TG analyzed the data. RU contributed the materials/analysis tools. GS, MR, and DK wrote the paper.

## Conflict of Interest

The authors declare that the research was conducted in the absence of any commercial or financial relationships that could be construed as a potential conflict of interest.
