# Supplementary material for: Replication in the Mononuclear Phagocyte System (MPS) as a Determinant of Hantavirus Pathogenicity
Source: Front Cell Infect Microbiol. 2020 Jun 12;10:281. doi: 10.3389/fcimb.2020.00281 (PMC7304325; doi:10.3389/fcimb.2020.00281)
Supplement: Supplementary file 1 [file Data_Sheet_1.pdf]

Supplementary Material

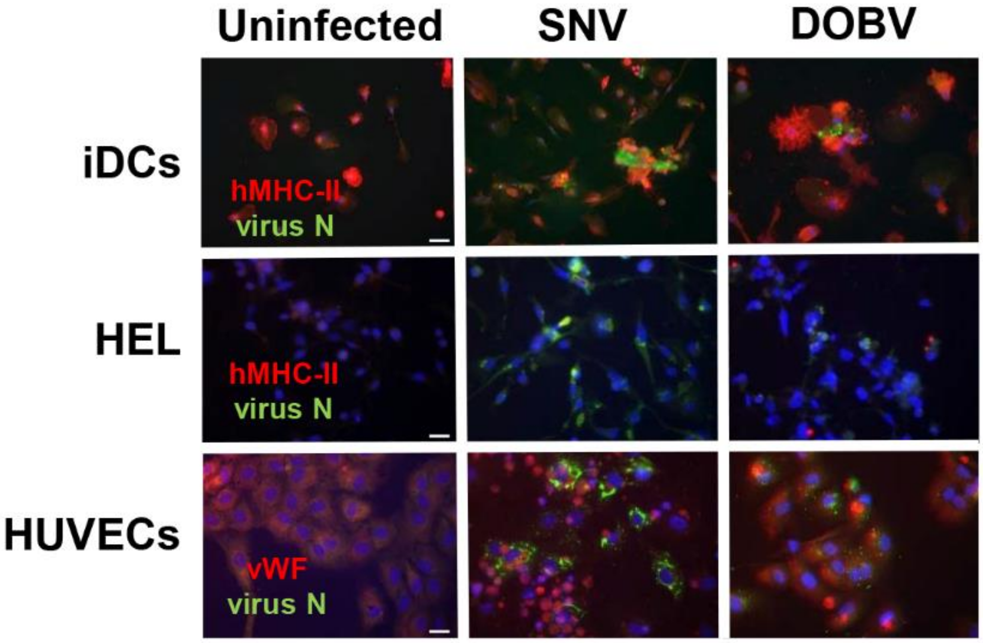

Figure S1 │ Susceptibility of myeloid cells to infection with Sin Nombre virus (SNV) and Dobrava-Belgrade virus (DOBV). Immature DCs (iDCs), human megakaryocyte cell line HEL stimulated with 10 nM PMA (HEL), and Human Umbilical Vein Endothelial Cells (HUVECs) were infected with SNV or DOBV (MOI of 1) for 4 days. Virus stocks were filtered by a 0.2 µm filter to remove possible debris that might be uptaken by phagocytosis. After 4 days cells were fixed, stained for hantavirus N protein (FITC, green), DNA (DAPI, blue), and either for MHC class II (Texas red, red) (Top and middle row) or the endothelial marker von Willebrand factor (vWF) (Texas red, red) (Bottom row) before immunofluorescence microscopy. Scale bar is 20 µm.

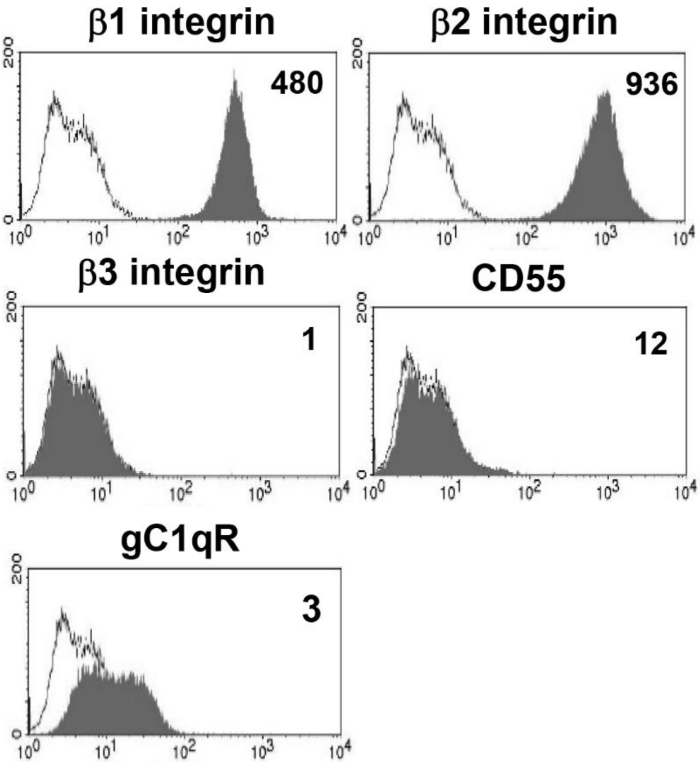

Figure S2 │ Expression of hantavirus receptors on immature DCs (iDCs). iDCs were stained for known hantavirus receptors (β1 integrin, β2 integrin, β3 integrin, CD55, gC1qR) and analysed by flow cytometry. Representative histograms are depicted. The x-axis shows staining with specific antibody (MFI in the upper right corner), the y-axis indicates cell count (open curve: isotype control).

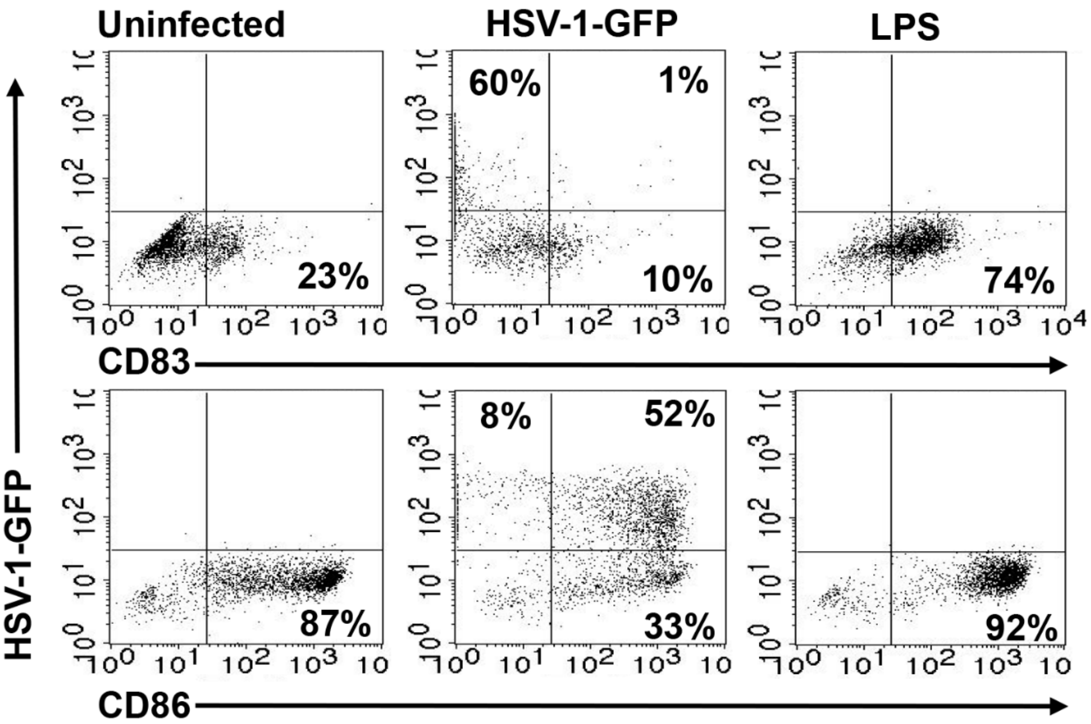

Figure S3│Downregulation of CD83 after infection of LADIII iDCs with herpes simplex virus type 1 (HSV-1*).* LADIII iDCs were exposed to GFP-expressing herpes simplex virus type 1 (HSV-1*)* strain KOS (K26G) for 24 hours. Subsequently, cells were stained for CD83 or CD86 and analysed by flow cytometry. The x-axis shows fluorescence intensity (log scale) of CD83 or CD86 expression, the y-axis depicts fluorescence intensity (log scale) of GFP expression. Percentage of gated cells is indicated in the relevant quadrants.

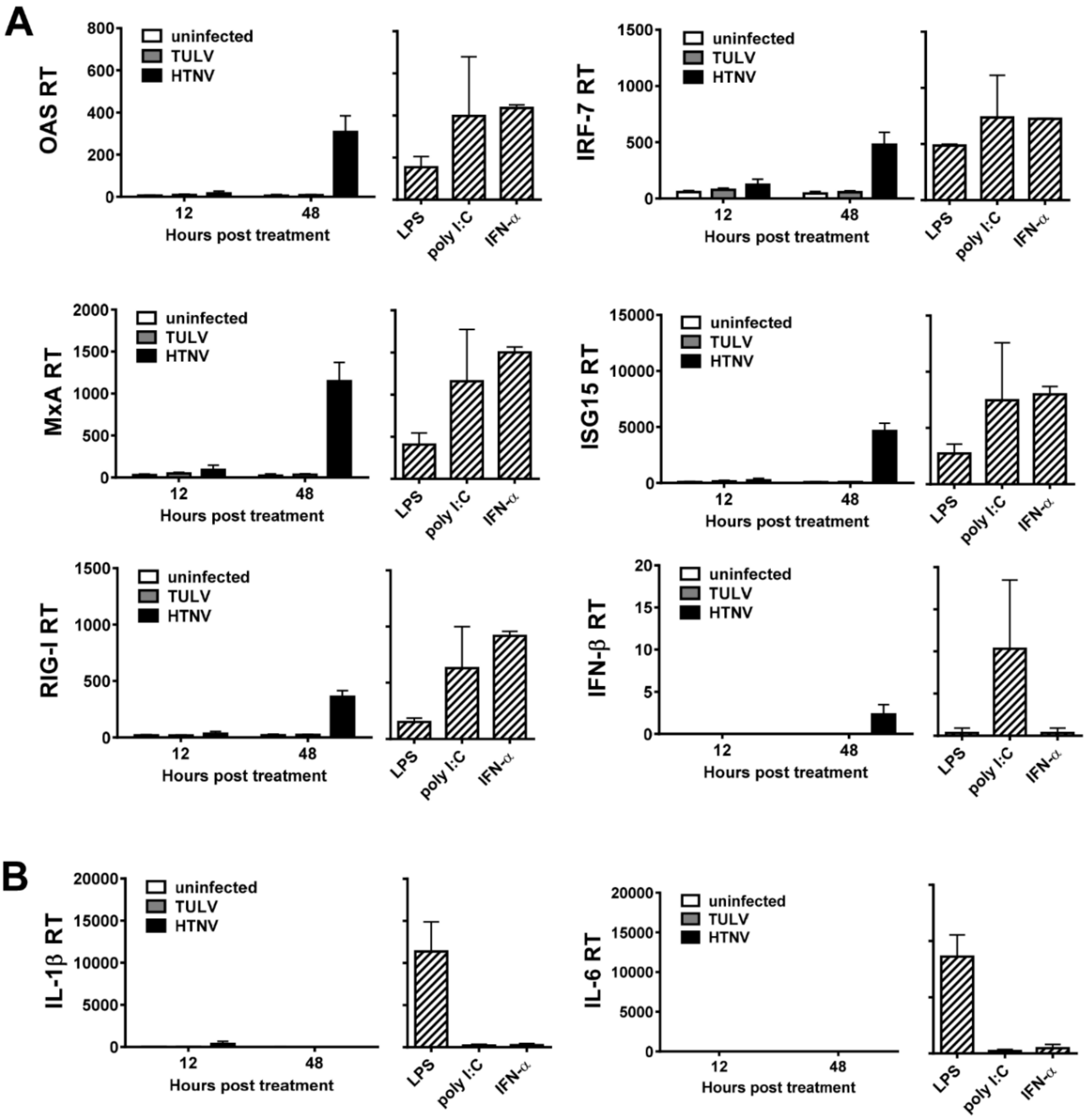

Figure S4 │ Comparative analysis of IFN-stimulated genes (ISGs) and NF-kB driven genes upon infection with HTNV or TULV. Comparative RT-qPCR analysis of human iDCs after infection with HTNV or TULV (MOI 1) for 12 hours and 48 hours, respectively. As positive controls cells were treated with LPS, poly I:C, or IFN-α 2a for 8 hours (dashed bars). **(A)** Expression levels of transcripts encoding ISGs (OAS, IRF-7, MxA, ISG15, RIG-I, and IFN-β). **(B)** Expression levels of transcripts encoding proinflammatory cytokines (IL-1β, IL-6). The data in **(A)** and **(B)** are shown as relative transcripts (RT). They represent means and SD of iDCs from three different donors.

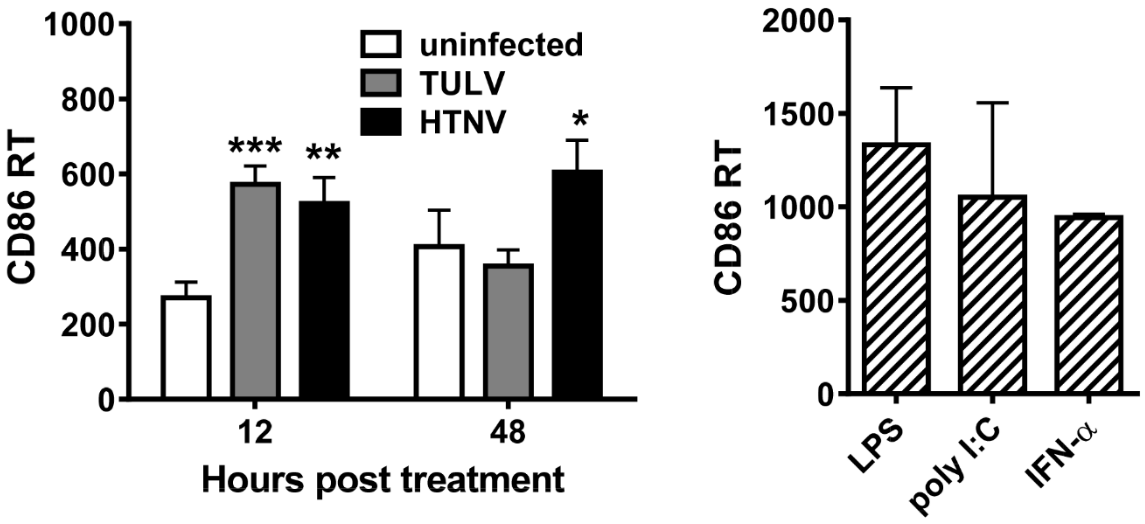

Figure S5 │ Increase in CD86 encoding transcripts after hantavirus infection of iDCs. iDCs were mock-infected or infected with HTNV or TULV (MOI 1.5) for 12 hours or 48 hours. As positive controls cells were treated with LPS (1 μg/ml), poly I:C (1 μg/ml), or IFN-α 2a (5000 U/ml) for 8 hours (dashed bars). The data of RT-qPCR analysis of CD86 transcript levels are shown as relative transcripts (RT) normalised to a housekeeping gene. Error bars represent the mean ± SD. The data are derived from iDCs from three different donors (*$p < 0.05$, **$p < 0.005$, ***$p < 0.001$; Students t test).
